# Spanish Validation for Olfactory Function Testing Using the Sniffin’ Sticks Olfactory Test: Threshold, Discrimination, and Identification

**DOI:** 10.3390/brainsci10120943

**Published:** 2020-12-07

**Authors:** María Luisa Delgado-Losada, Alice Helena Delgado-Lima, Jaime Bouhaben

**Affiliations:** Experimental Psychology, Cognitive Processes and Speech Therapy Department, Faculty of Psychology, Complutense University of Madrid, Campus de Somosaguas, 28223 Pozuelo de Alarcón, Spain; alicedel@ucm.es (A.H.D.-L.); jaimebou96@gmail.com (J.B.)

**Keywords:** validation, cultural adaptation, olfaction, smell, olfactory threshold, olfactory discrimination, olfactory identification, Sniffin’ Sticks

## Abstract

The assessment of olfactory function is becoming increasingly relevant, especially in cases of cognitive decline (i.e., neurodegenerative diseases), where olfactory alterations may be relevant as potential early biomarkers. The Sniffin’ Sticks Olfactory Test, developed in Germany and validated in several countries, is an objective measure of olfactory performance. This study aims to validate this test in a Spanish sample. This study included 209 healthy normosmic volunteers (154 females and 55 males) aged between 20 to 79 years (mean age = 50.11 ± 15.18 years) as the normative sample. From this group, 22 participants were retested in order to obtain test–retest reliability evidence. Odor familiarity for descriptors in the olfactory identification test was also studied on an independent healthy sample (*n* = 69), and required cultural modifications were applied. Results indicate that men and women, as well as smokers and non-smokers, performed equally in every test. However, significant differences were found between age groups in every score. The general trend is that olfactory function progressively decreases as a function of age, the elderly group (+60 years) being the one with the lowest scores. In conclusion, this normative data, in addition to the test’s cultural modifications, allows the Sniffin’ Sticks Olfactory Test to be administered on a Spanish population.

## 1. Introduction

Sense of smell, responsible for detecting and processing odors, is one of the oldest and most important senses for living organisms. It provides critical information about our surroundings [1]. Smell can influence our emotional state, cognition, and behavior [2,3]. From birth to adulthood, our sense of smell regulates many of our behaviors, from nutrition to social interaction [4]. Smells connect us with moments in our past, alert us, cause us to flee or attack, and revive feelings and emotions [5]. Smells are not perceived in the same way by all people; they vary in intensity and magnitude. The olfactory function is influenced by climate [6], age [7,8,9], gender [10,11], and culture [12,13,14].

Olfactory dysfunction is a common problem to which less attention is usually paid because it is considered to have a less serious clinical implication, as the alteration of smell is less disabling than alteration in one’s sight or hearing. Because smell disorders are rarely fatal, patients often do not receive adequate medical care. However, an impaired sense of smell can have a negative effect on quality of life and safety, and can be a sign of other health problems [15,16,17].

While vision and hearing have been treated as basic senses for general health, smell is gaining increasing interest in clinical and research settings, as more and more pathologies are associated with altered smell. Some examples are diabetes mellitus [18,19], rhinitis, sinusitis [20,21], anxiety [22,23], major depression [24,25,26,27], schizophrenia [28], autism [29], liver disease [30,31], arterial hypertension [32], and neurodegenerative diseases [33,34], such as frontotemporal dementia [35,36,37] and amyotrophic lateral sclerosis [38]. More recently, a plethora of scientific evidence indicates that deterioration of smell is a biological marker in the preclinical phase of neurodegenerative diseases, such as Parkinson’s disease [39,40,41,42] or Alzheimer’s disease [43,44,45].

There is a full consensus stating that sense of smell gradually decreases with age, especially after the age of 60 [8,9,46,47,48,49,50,51,52,53,54,55], but in relation to sex, the disagreement between studies is pronounced [10,11,54,55,56,57,58]. There is also no consensus to indicate that smoking accelerates this loss [50,59,60].

Olfactory testing is comprised of three different components: olfactory threshold, odor discrimination, and odor identification. The olfactory threshold represents the level of detection of odors at low concentrations, odor discrimination is the non-verbal distinction between different odors, and odor identification represents the ability to name or associate an odor.

There are different ways to assess nasal chemosensory performance. This is typically done by asking the patient to identify common food or essential oil odors, or even just using the patient’s subjective information. Assessing only the ability to identify odors, although useful for detecting severe olfactory loss (anosmia), is insufficient to detect lower degrees of loss (hyposmia) or to discriminate between olfactory and trigeminal stimulation. Having more extensive instruments that allow us to quantitatively and reliably assess olfactory capacity, make accurate diagnoses, evaluate prognoses, and monitor and evaluate the effect of treatments is essential for clinical and research.

One such objective and quantitative measurement of olfactory function is the Sniffin’ Sticks Olfactory Test. The Sniffin’ Sticks Olfactory Test is an optimal performance test that evaluates these three components of olfaction to generate a composite score resulting from the summation of threshold, discrimination, and identification.

The Sniffin’ Sticks Olfactory Test was designed and validated over 20 years ago [61]. Since its first publication, versions of the test have been made with modifications of odors [62,63,64], as well as extended [65,66] and abbreviated [67,68,69] versions. Furthermore, it has been adapted and validated in Asia [70,71,72], Australia [73], and in various European countries, such as Romania [74], Italy [75], Greece [76], Portugal [77], Holland [78], the United Kingdom [79], Turkey [80], and Denmark [81,82]. However, to our knowledge, normative data on the Spanish population has not yet been published.

Odor identification is affected by cultural differences because it is based on the individual’s familiarity with the test odorants and descriptors. Cultural aspects have a large impact on the exposure and frequency of foods and smells, and consequently on familiarity with smells. To obtain an accurate assessment of olfactory function with the test, participants need to be familiar with all of the descriptors that are used. Adaptation of odorants and distractors in the cultural setting where the test is applied is necessary [76,77]. The validity of identification tests has been examined both in terms of internal validity [61,77,83] and external validity [71,72,76,77] in other languages and cultures. Using the unadapted version of the identification test can potentially diagnose hyposmia in an individual with a normal sense of smell [76].

All published studies, both those carried out with smaller groups of participants and those carried out with a large number of participants [83,84,85], have demonstrated the usefulness of the Sniffin’ Sticks Olfactory Test to evaluate olfaction in different populations from all over the world, but there are no similar data for a population in Spain.

Having normative data on this test would allow its application in diagnosing, studying, and measuring the efficacy of treatments on multiple olfaction pathologies in the Spanish population. We decided to adapt and validate this olfactory test and not others due to its European cultural origin, and because it allows the three components of smell to be studied (threshold, discrimination, and identification), instead of just identification, which is the case with other tests. For example, the University of Pennsylvania Olfatory Identification Test (UPSIT) [86] evaluates the identification capacity through 40 odorants impregnated in paper strips. The odorants are liberated by scratching microencapsulated odor labels mounted on paper. The participant has to scratch the paper and select one in four names for the selected odorant.

Unlike UPSIT, the Connecticut Chemosensory Clinical Research Center (CCCRC) [87] consists of a threshold and a supraliminal subtest with eight encapsulated odorants in cans that the participant has to identify. The patient has a list of 20 odors that can be consulted to indicate each of the eight odorants presented. Both tests are widely used in the United States.

In Europe, the Scandinavian Odor Identification Test (SOIT) [88] has been developed, which evaluates the identification performance of 16 odorants presented in 80 mL glass bottles. For each stimulus, the participant is provided with a written list of four response alternatives from which to choose the most appropriate item for identification. Another olfactory test is the Smell Diskettes Olfaction Test (SDOF) [89], which consists of eight samples containing different odorants in plastic containers. The samples are opened to release the odor, and the participant smells and identifies which odor it is. This test is designed as a multiple choice forced triple test.

None of the abovementioned tests (UPSIT, CCCRC, SOIT, or SDOF) have been adapted or validated in as many countries as the Sniffin’ Sticks Olfactory Test, and none of them allows testing of the three components of olfactory performance to be evaluated in such a precise, valid, and reliable way.

The objective of this work is to adapt the Sniffin’ Sticks Olfactory Test to the Spanish population through the development of normative scales, including evidence of reliability, as well as the study of potential demographic covariates, such as sex or age, that could be related to olfactory capacity. Hence, within this objective, the study design is composed of three sections: (i) cultural adaptation of OI test, as the percentage of familiarity with each odor descriptor; (ii) analyses of covariates for each measure and description of standardized values; and (iii) study of test–retest reliability.

## 2. Method

### 2.1. Participants

The complete version of the Sniffin’ Sticks Olfactory Test (TDI) was applied to an initial pool of 242 participants aged between 18–79 years old. These participants were enrolled from social media, advertisements in public places (such as universities, libraries, and contact with private companies), and from Hospital Central de la Cruz Roja (Madrid, Spain). Assessments took place between July 2020 and September 2020. Inclusion criteria were: (i) to be 18 years or older, (ii) absence of current otorhinolaryngology alterations, and (iii) compliance with testing procedure. Exclusion criteria included: (i) medical history of olfactory alterations, including nasal polyposis, sinusitis, or prior nasal surgery, (ii) to have reported COVID-19 compatible smell symptomatology, (iii) presence of nasal congestion at the moment of test administration or recent upper respiratory tract infection within two weeks, (iv) medication intake with repercussion in olfactory performance (such as some antibiotics, antiepileptics, antithyroids, benzodiazepines, or antiarrhythmics), (v) presence or suspicion of cognitive impairment and/or neurologic or psychiatric dysfunctions, and (vi) pregnancy. From the sample, 22 participants completed a second administration of the TDI Test approximately two weeks after the first one in order to study test–retest reliability.

Alongside the Sniffin’ Sticks Olfactory Test and in order to check eligibility criteria (inclusion and exclusion), participants were administered a brief questionnaire, based on the Multilingual International Questionnaire [90], in order to collect information about demographic and clinical variables and data related to health behaviors (smoking, alcohol consumption).

Another independent sample of 80 participants was contacted via online media in order to complete a questionnaire about the familiarity with the odor descriptors from the identification subtest. They were also asked to complete a brief questionnaire about their current olfactory function, including a self-rating olfactory scale from 0 to 10.

### 2.2. Measures

The Sniffin’ Sticks Olfactory Test (Burghart GmbH, Wedel, Germany) is an olfactory test that intends to objectively measure nasal chemosensory performance [61,83]. The complete version includes three tests that aim to measure different components of olfactory function, namely, olfactory threshold (OT), odor discrimination (OD), and odor identification (OI). Each test gives a unique score, ranged from 0 to 16, representing each olfactory component, and it may also be administered independently. The sum of the three odors scores (OT, OD, and OI) defines a composite score (TDI, ranged from 0 to 48), which measures general olfactory function. Previous work has already established the test–retest reliability and its validity in comparison with established measures of olfactory sensitivity [91].

The OT test consists of 16 triplets of pens, where, in each triplet, there is one pen with an odorant and the other two with a solvent distractor. The odorant is equal in each triplet; it only varies in its concentration. In each trial, the pens of the triplet were presented in a randomized order, obtaining the answers through a three-alternative forced-choice method. Participants have to identify the odor-containing pen in each triplet, being the triplet presentation mode by ascending concentration. When participants score two consecutive correct answers, another set of three pens with a one-step lower concentration is presented. The OT score is defined by the procedure of Hummel et al. [61]: as the mean of the last four of seven staircase reversal points.

The OD test consists of a set of 16 triplets of odor pens: two with the same scent (distractors) and the other one, the target, with a different scent. Each triplet composes a three-alternative forced-choice task: participants have to identify the pen with a different odor from the other two. The interval between triplets’ presentations of the odors is 20–30 s.

The OI test is composed of a set of 16 pens, each one presenting a different and identifiable scent (i.e., apple, orange, coffee, etc.). Using a four-alternative forced-choice method for each pen, participants have to smell the pen and choose an answer from a list of four verbal items. The interval between presentations of each odor is approximately 20 s.

The combined result of the three subtest were presented as a composite score (TDI), which was derived from the sum of the results obtained for the threshold, discrimination, and identification measures.

### 2.3. Testing Procedure

Prior cultural adaptation of identification test: OI subtest employed a four-alternative forced choice for each item, as was said before. This modality of odor identification is tied to cultural bias, since it is related to the participant’s familiarity with the odor target and its distractors. Hence, multiple-choice answers from the OI subtest require a cultural adaptation to the area where it is planned to be administered. The current study includes a cultural adaptation of the identification test to the Spanish population in order to assure odor familiarity with OI descriptors and to minimize potential risks and biases due to a lack of familiarity with these.

The exact translation of the odorant descriptors and distractors was done using the established forward-backward procedure. Two independent bilingual (English and Spanish language) health professionals performed the translation from English to Spanish language. Two different bilingual health professionals then translated the provisional Spanish version back into English. The final version was comparable to the original version. As several Spanish translations were found for various odor descriptors, familiarity with these odor descriptors was measured in a Spanish native sample. This procedure follows a similar the methodology established by Ribeiro et al. [77].

Sniffin’ Sticks Olfactory Test administration. The administration procedure follows the one which was established in the original version [61]. The order of test presentation is threshold, discrimination, and identification. Olfactory function was assessed for both nostrils. For odor presentation, pens with a length of 14 cm and a diameter of 1.3 cm were used. Each pen was filled with 4 mL of the corresponding liquid odorant. The evaluator took the pen’s cap off and put the tip of the pen in front of the participant’s nostrils, with an approximate distance of 2 cm. In any case, the tip of the pen physically touched the participant’s nose. In all of the three tests, each odor pen was presented to the participant for 3 s. The overall time of administration ranged from 30 to 45 min, depending on how long the OT subtest lasted.

Testing of participants was performed in a quiet, well-ventilated room to avoid any background smell interfering with the test odors and with the use of odorless gloves. All participants were told not to eat, drink, smoke, chew gum, put on cologne, or brush their teeth up to 1 h before participating in the test (they could drink water).

## 3. Experiment Design

The study was ruled by the principles of the Declaration of Helsinki (Edinburgh, 2013) and was approved by the ethics committee from the University Hospital San Carlos (Madrid, Spain) (ref. number: 20/515-E). All participants who were administered the Sniffin’ Sticks Olfactory Test signed an informed consent. Participants who were online polled agreed with their participation by answering the online survey.

Three experiments were included in the study protocol.

**Experiment 1. Cultural adaptation.** Odor familiarity was measured on the translation of the original 48 descriptors, plus three translation choices (51 in total). With the objective of determining this familiarity, an online survey was administered to 80 participants from Complutense University of Madrid. Participants were asked to rate odor descriptors according to the familiarity degree they thought each odor had with a Likert scale ranging from 1 (not familiar) to 5 (very familiar). They were also asked demographic (sex, age) and olfactory questions (COVID-compatible olfactory symptomatology, history of otorhinolaryngology alterations, self-rating of olfactory function from 0 to 10). The final sample after data cleaning (participants who reported olfactory alterations or whose self-rated olfactory function was below five points) was composed of 69 participants (15 males and 54 females), who agreed to answer the online survey and were aged between 21 and 79 years (mean = 48.46, SD = 21.4). The cutoff point of five in the self-rated olfactory function scale (from 0 to 10) covers the range in the scale (from 5 to 10 points), which represents a positive subjective perception of olfactory function. As familiarity data was obtained through an online survey, this criterion was established due to the unavailability of other clinical data.

**Figure 1:**
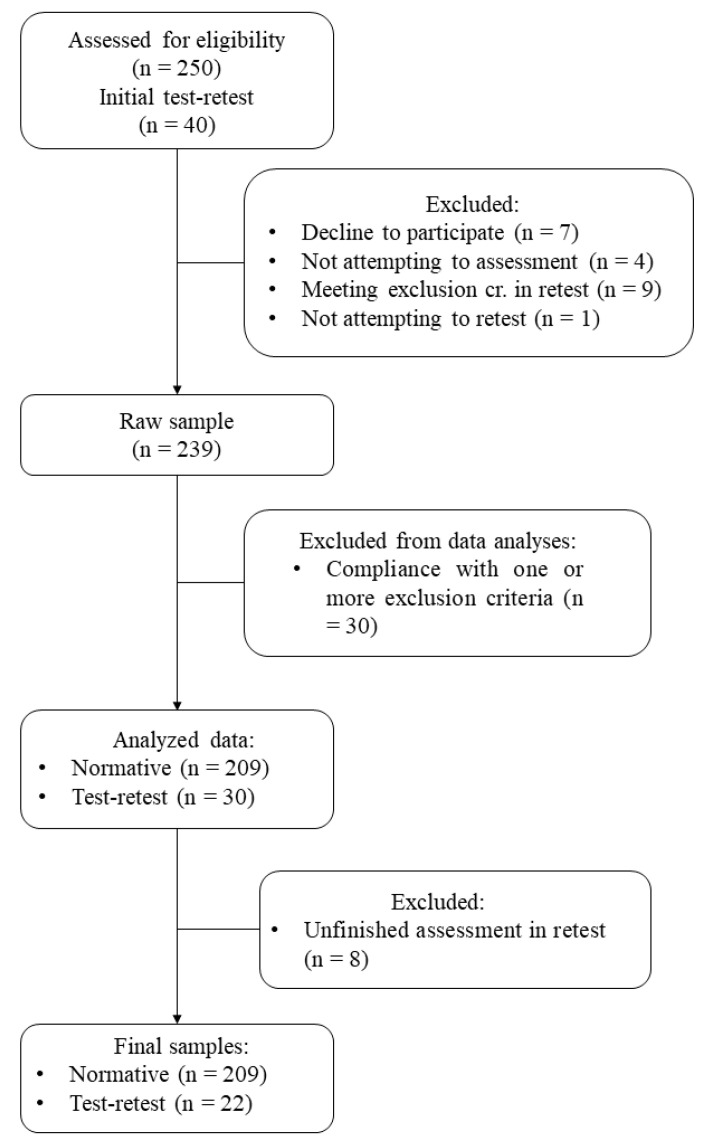
Participants’ flow chart. Source: elaboration based on the information obtained.

**Experiment 2. Normative sample.** The complete version of the Sniffin’ Sticks Olfactory Test was administered to 242 participants. This complete version was composed of OT, OD, and OI (blue part) subtests. After outlier detection and data cleaning, normative values were calculated for a final sample of 209 participants (154 females, aged from 18 to 79 years, mean age = 50.11 years, SD = 15.18, and a mean self-rated olfactory function of 6.96 (SD = 1.75) out of 10)). The participants’ flow chart for the normative sample is shown in Figure 1.

**Experiment 3. Test–retest reliability and internal consistency.** Forty participants from the normative sample (*n* = 209) were intended to be retested approximately two weeks after the first test administration. The final retest sample was composed of 22 participants. In any case, the test–retest interval was longer than four weeks. Statistics for test–retest reliability were computed within this sample. Internal consistency was studied in the complete normative sample (*n* = 209).

## 4. Statistical Analyses

The whole statistical analysis plan was performed with R software, version 3.5.2 [92]. For significance testing, the alpha level was set to 0.05 (α = 0.05).

Regarding the cultural adaptation sample (*n* = 69), the mean and standard deviation for age and self-rated olfactory function and female proportion for sex were obtained. Then, ratings for each odor descriptor were averaged and transformed to a percentage scale (where 5 from the Likert scale equals a 100% familiarity) in order to enhance results interpretation. This transformation of the Likert scale to percentage of familiarity is covered by the methodology of Ribeiro et al. [77]. The cutoff point of 75% familiarity covers Likert choices 4 (quite familiar) and 5 (very familiar), while scores greater or equal than 50% familiarity covers choice 3 (familiar).

Descriptive analysis was firstly performed over the complete normative sample. This descriptive analysis included outlier detection and data deleting due to exclusion criteria. Afterwards, multiple linear regression analyses were adjusted (under the ordinary least squares method) on each test score (OT, OD, OI, and TDI) as the dependent variable, including age, sex, and smoking status as potential covariates. The stepwise method was chosen in order to remove non-significant predictors from the regression model. Pairwise *t*-test comparisons between covariates’ categories were intended for each statistically significant (*p* < 0.05) predictor, under the false discovery rate correction method [93] for test multiplicity. For normative table, data were summarized in count, mean, standard deviation, 95% confidence interval of the mean, minimum and maximum, and 5, 10, 25, 50, 75, 90, and 95 percentiles.

**Figure 2:**
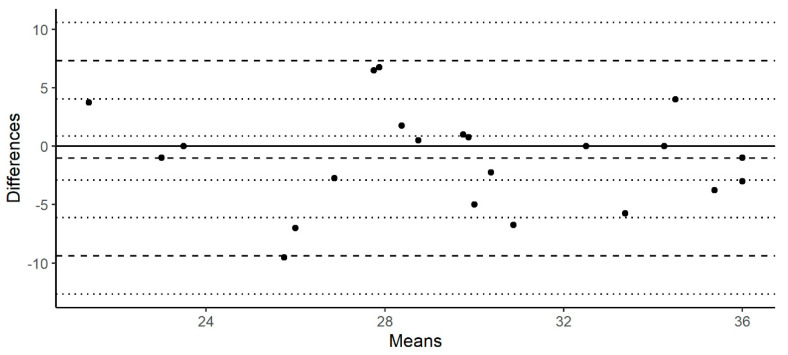
Bland−Altman plot for the test−retest TDI score.

Regarding the test–retest reliability and internal consistency, mean concordance, intraclass, and Pearson correlation coefficients were computed for each score within the test–retest sample (*n* = 22). The intraclass correlation coefficient was estimated following a two-way model for single units and based on the consistency of responses. The mean concordance coefficient was calculated following the procedure of Lin [94]. Bland–Altman plots were generated for the TDI score (Figure 2). Cronbach’s alpha and Spearman–Brown coefficients were calculated on the normative sample (*n* = 209) in order to assess the internal consistency of each score.

## 5. Results

Odor familiarity was rated with a 1–5 Likert-type scale by a panel of 69 participants (15 males and 54 females aged between 21 and 79 years (mean = 48.46, SD = 21.4)). All ratings per item were averaged and transformed to a percentage scale, which aimed to measure the percentage of familiarity. Table 1 shows the percentage of familiarity for each odor descriptor.

More than half of odor descriptors (27/51) show familiarity percentages above 75%, but the familiarity of almost all of the odor descriptors (46/51) was above 50%. With the described familiarity results, the original odorants contained within the pens were unchanged, but the descriptors were replaced by terms more familiar to Spanish speakers. It was decided to change the translation of the following odor descriptors: trementina (trementine, % familiarity = 42.61) with disolvente (solvent, % familiarity = 79.125), chucrut (sauerkraut, % familiarity = 37.97) with coles (% familiarity = 50.72), and camomila (camomille, % percentage = 47.67) with manzanilla (% percentage = 66.38). It was decided not to change the remaining one, whose familiarity score is under 50% (caucho, rubber) due to the lack of a more suitable semantic descriptor, as stated by the adaptation supervisors.

Descriptive statistics in the overall normative sample are shown at the top of Table 2. Then, a multiple linear regression analysis was performed over each one of the four smell measures (OT, OD, OI, and TDI), including sex, age, and smoking status as potential covariates. Only a statistically significant main effect of age as a predictor was found in OT (*r* = −0.412, *b* = −0.072, *p* < 0.001), OD (*r* = −0.294, *b* = −0.047, *p* < 0.001), OI (*r* = −0.241, *b* = −0.033, *p* < 0.001), and TDI (*r* = −0.491, *b* = −0.153, *p* < 0.0001). Normative values are shown in Table 2. Due to the effects of age as a predictor, it was decided to categorize this variable in five groups: twenties (20−30), thirties (30−40), forties (40−50), fifties (50−60), and elderly (>60) in order to generate the normative table. Although there is not enough evidence to assume sex differences in olfactory performance, descriptive statistics of OT, OD, OI, and TDI scores per sex and age groups are reported in Supplementary Table S1.

**Figure 3:**
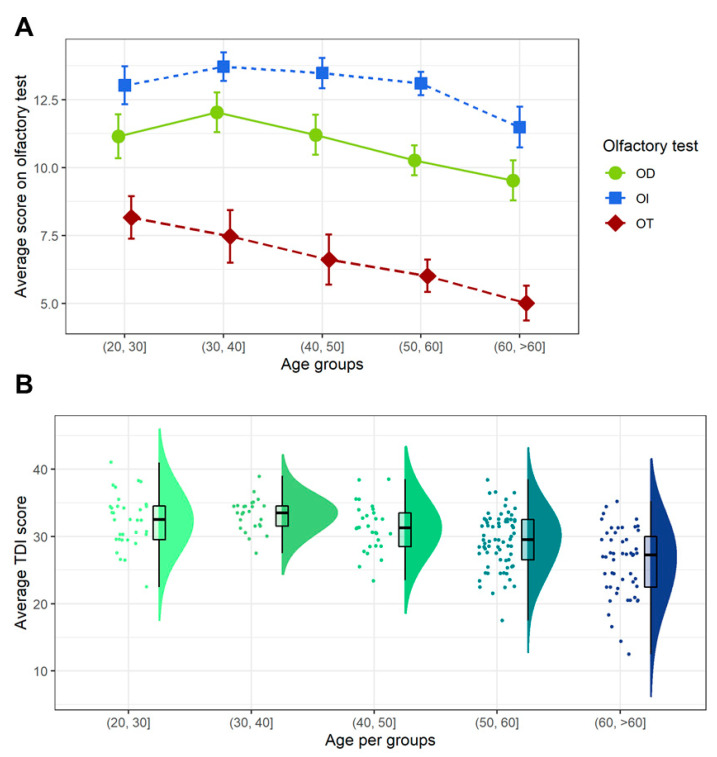
Graphical representation of Sniffin’ Sticks Olfactory Test scores by age groups. (**A**) OT, OD, and OI scores. (**B**) TDI score.

Pairwise independent t-test comparisons between age groups were calculated for each olfactory measure. Results for these multiple comparisons, under false discovery rate correction [93] of the *p*-values, are as follows (negative t statistic indicates that the difference favors the younger group). In the first place, the twenties group performed better in OT scores than the forties (*t* = −2.486, *p* = 0.023), the fifties (*t* = −4.153, *p* = 0.0002), and the elderly groups (*t* = −5.743, *p* < 0.0001), but no better than the thirties group (*t* = −1.068, *p* = 0.287). The thirties group showed a higher score than the fifties (*t* = −2.544, *p* = 0.023) and the elderly group (*t* = −4.096, *p* = 0.0002), but there was no evidence of superior performance compared to the forties group (*t* = −1.277, *p* = 0.253). No statistically significant differences were found between the forties and the fifties groups (*t* = −1.102, *p* = 0.287). Both the forties (*t* = −2.8, *p* = 0.014) and the fifties (*t* = −2.224, *p* = 0.039) groups performed statistically higher than the elderly. Regarding with OD score, the twenties group performed significantly better than the elderly group (*t* = −3.073, *p* = 0.006), but no differences were found between the youngest group and the thirties (*t* = 1.418, *p* = 0.197), forties (*t* = 0.092, *p* = 0.928), and fifties (*t* = −1.776, *p* = 0.129) groups. The thirties scored higher than the fifties (*t* = −3.226, *p* = 0.006) and elderly (*t* = −4.353, *p* = 0.0002) groups, but not the forties (*t* = −1.292, *p* = 0.23). The difference favoring the forties group over the elderly one was also significant (*t* = −3.053, *p* = 0.006). No differences were found either between forties and fifties (*t* = −1.804, *p* = 0.129) or between fifties and elderly groups (*t* = −1.702, *p* = 0.129). In relation to OI, the twenties group only scoreed statistically higher than the elderly (t = −3.365, *p* = 0.002), but no higher than the thirties (*t* = 1.27, *p* = 0.343), forties (*t* = 0.868, *p* = 0.495), or fifties (*t* = 0.158, *p* = 0.874) groups. The thirties group also performed significantly better than the elderly group (*t* = −4.458, *p* = 0.0001), but not in comparison with the forties (*t* = −0.424, *p* = 0.746) and fifties (*t* = −1.304, *p* = 0.343) groups. The forties group showed a significantly higher score than the elderly group (*t* = −4.182, *p* = 0.0001), but this score was no different from the fifties one (*t* = −0.851, *p* = 0.495). Scores from the fifties group were higher than the elderly (*t* = −4.275, *p* = 0.0001). Finally, the twenties group showed higher performance in the TDI global score than the fifties (*t* = −3.412, *p* = 0.0001) and elderly (t = −6.85, *p* < 0.0001) groups. The thirties group also performed significantly better than the fifties (t = −4.009, *p* = 0.0001) and elderly (*t* = −7.143, *p* < 0.0001) groups. The forties group scored statistically higher than the elderly group (*t* = −5.49, *p* < 0.0001), but no higher than the fifties one (*t* = −2.111, *p* = 0.051). The fifties group also had a better performance than the elderly group (*t* = −4.421, *p* < 0.0001). There was not enough evidence to assume a statistical difference between twenties and thirties (*t* = 0.805, *p* = 0.421), twenties and forties (*t* = −0.996, *p* = 0.356), or thirties and forties (*t* = −1.711, *p* = 0.11) in the TDI global score. Figure 3A shows the visualization of the three smell tests (OT, OD, and ID), and Figure 3B shows the visualization of the global score, both as a function of age.

The sample from experiment 3 (*n* = 22) was retested in order to assess the test−retest reliability of the instrument. The mean concordance, intraclass, and Pearson’s correlation showed moderately proper values (>0.5, according to Koo & Li criteria [95]) in OI and TDI scores. The test−retest reliability was under 0.5 in OT and OD measures, but values for both ranged from 0.45 to 0.5. The Bland−Altman plot for the TDI score is shown in order to complement test interpretation (Figure 2). Cronbach’s alpha and the Spearman−Brown coefficient were calculated in order to assess the internal consistency of each smell measure. Values for these statistics (<0.7) do not allow to state that the test’s scores show a proper internal consistency [96]. Test−retest reliability and internal consistency results are reported in Table 3.

## 6. Discussion

To our knowledge, this work presents the first validation of the Sniffin’ Sticks Olfactory Test in the Spanish population. Around 38 million people over the age of 20 now have access to a smell test adapted and scaled to Spanish culture useful for diagnosing olfactory disorders. The validated test can be used not just in Spain, but also abroad or in other regions that share the same language and culture.

The Sniffin’ Sticks Olfactory Test is an olfactory test recommended by various national and international medical societies of otorhinolaryngology as a standard for olfactory tests [61]. This instrument has the advantage of allowing the three components of smell (threshold, discrimination, and identification) to be studied, while other tests only evaluate one of the components.

Olfactory assessment tests have important cultural components [12,14,71,74,76,77,97]. In this study, we have tried to solve the difficulties derived from the factors of cultural bias by adapting the descriptors used in the odorants and distractors applied.

The original odorants contained in the sticks were not modified, but the results obtained in the applied familiarity survey indicated the need to replace some descriptors (trementina, churcrut, and camomila—terpentine, sauerkraut, and chamomile) with more common terms in Spanish (disolvente, coles, and manzanilla), respectively. The modifications made do not imply changes in the test’s application.

This study presents the normative data for the evaluation of olfactory capacity using the Sniffin’ Sticks Olfactory Test in a Spanish population. The normative data are presented in tables to be used as a guide to estimate individual olfactory capacity in relation to the individual’s age. The tables allow us to compare the performance of people over 20 years old, assigning a range of deciles compared to their peers of a similar age. The decision about this age categorization by 10 years was made based on the intention to capture olfactory differences across the lifespan. Sample sizes per age group are similar to the ones in other normative studies with wider intervals [6,71].

The TDI score at the 10th percentile was 27.8 in the younger participants, 30.2 in the thirties group, 27.3 in the forties group, 24.5 in the fifties group, and 20.5 in the elderly group. The 10th percentile has been used to discriminate between normosmic and hyposmic people. These values in the 10th percentile are similar to those found in other validation studies [71,82,97].

All OT, OD, OI, and TDI scores were inversely associated with age. This age-related change in olfactory sensitivity has been described in numerous previous studies [8,47,48,49,50]. Our results indicate a less efficient performance in all olfactory tests from the age of 50 onwards. The olfactory threshold is the component in which the greatest changes are observed in relation to age, compared to the discrimination and the identification of odors. These results are consistent with those obtained in studies conducted in all countries that have developed normative values for the Sniffin’ Sticks Olfactory Test for different age groups [70,71,72,73,74,75,76,77,78,79,80,81,82].

A single cause has not been identified for the findings related to the effect of age on olfactory ability. Some authors argue that age-related olfactory disorders may be due to changes in neural and cortical pathways or changes in cognitive abilities, such as attentional or memory difficulties that may influence the ability to recognize, remember, and associate [98] and, therefore, affect the processing of olfactory information [51,99]. Others consider that it occurs due to changes that take place in the anatomy of the nasal cavity or in the neuroepithelium, changes in the olfactory epithelial blood flow, decreased metabolic activity, increased viscosity of mucus, or obstruction of the cribriform plate that occurs naturally with aging [54,100,101,102,103,104].

The presence of olfactory alterations early in the course of neurodegenerative diseases suggests that systematic clinical evaluation of this function can provide valuable information for early detection [43,44,51,105]. The possible relationship of olfactory dysfunction to Alzheimer’s dementia has been suggested in a number of research findings [106,107,108,109]. The impaired olfactory function may be a marker of the conversion from mild cognitive impairment to Alzheimer’s dementia [110,111,112,113], and has been cited as predictive for the development of mild cognitive impairment in healthy controls [114,115,116]. In addition, other studies have also reported significant deficits in the smell functioning of people with possible and probable Alzheimer’s disease [117,118,119]. For this reason, it is important to extend the use of smell assessment tests as part of the neuropsychological examination in adult and elderly people with subjective complaints due to loss of cognitive abilities.

No statistically significant differences were found in terms of sex in any of the Sniffin´ Sticks Olfactory Test subtests, as in most of the test validation studies in different countries [10,73,76,77,80,84]. Although there is open discussions about gender performances in olfactory tests, it is accepted that women perform better in olfactory tests due to hormonal factors, such as oestrogens present in the female olfactory epithelium. In this investigation, such differences were not found; this might be due to the high percentage of female participants in this study, which might be blurring such distinctions [74,82,84,85].

No differences were found between smokers and non-smokers, similarly to other validation studies of the smell test where this condition was also considered among the participants [76,82,97]. Regarding the influence of smoking, in the study of Landis, Konnerth and Hummel [120], 20% of the participants were active smokers, and still no differences were found in olfactory performance between smokers and non-smokers in age-controlled comparisons. Our results are also in line with the investigation of the Danish validation of the Sniffin’ Sticks Olfactory Test where the smoking habits were controlled in three different groups: active smokers, former smokers, and never smokers, and no statistical differences were found [82].

In the present study, we analyzed the internal consistency and reliability of the different subtests. The correlation statistics used to assess test−retest reliability show moderately adequate values (>0.5) in identification [94], and are close to this criterion in thresholds and discrimination. This differs from the internal consistency statistics used (<0.7). Although the reliability and internal consistency of the subtests has been found adequate in some studies [77], since the initial validation of the test low values can be seen in the test−retest reliability, especially in the threshold test [61]. Although test−retest reliability values in our study are moderate, other validation studies with larger sample sizes, such as the ones from Taiwan [71] with 42 participants or from Portugal [77] with 71 participants, show that the Sniffin’ Sticks Olfactory Test is reliable and stable. Hence, further studies in the Spanish population should notice this point and increase test−retest sample sizes. However, trends in our values indicate proper test reliability if sample size increases.

While this study has a number of strengths and makes an important contribution by making normative data available to clinicians and researchers from a widely used smell assessment test in Europe and other continents, it also has limitations. With an eye on future studies, we consider the need to replicate this research with a larger number of participants, and it would also be in our interest to balance the proportion between female/male participants in order to have a clearer view of the role that gender plays on olfactory performance. It could be the case that some of the subgroups encompass a significant sociocultural heterogeneity, or they may have limited knowledge of or previous exposure to the odors used in the test, and the lack of familiarity may influence the performance obtained in certain odorants and the test among all participants. This highlights the need for future efforts to be aimed at alleviating these aspects, and validating the test in larger and more heterogeneous population groups. The application of the test in patients and controls is important to be able to determine the specificity and sensitivity of the test, as well as to evaluate the construct validity using the version culturally adapted to people with a reduced sense of smell.

## 7. Conclusions

This study provides different normative data for each of the age groups. The Sniffin’ Sticks test is a suitable tool to evaluate olfactory capacity in the clinical and research environment.

It is necessary to have an adaptation that eliminates possible errors due to cultural factors in the odor identification test, such as the one carried out in this work.

The results do not indicate that there is a relationship between smell and sex, or between smell and smoking. However, changes in olfactory function are observed as age progresses, with a more pronounced decrease after 60 years of age.

This decrease is probably due to a series of factors, predominantly epithelial alterations, blood disorders, or the increase in the viscosity of the mucus associated with age, so it would be useful to extend the use of the term “presbyopia” as the gradual decrease or loss of smell as a consequence of aging. The usefulness of this concept would contribute differences with respect to the beginning of a possible deterioration in the olfactory capacity associated with the development of neurodegenerative diseases or other pathologies.

## Figures and Tables

**Table 1:** Familiarity percentage of OI answer options.

Original Odor Descriptor	Proposed Spanish Translation	% Familiarity	Original Odor Descriptor	Proposed Spanish Translation	% Familiarity
Orange	Naranja	97.101	Cookies	Galletas	77.971
Coffee	Café	95.942	Coconut	Coco	76.522
Cheese	Queso	93.623	Licorice	Regaliz	74.203
Onion	Cebolla	93.043	Menthol	Mentol	73.913
Fish	Pescado	93.043	Pepper	Pimienta	73.043
Lemon	Limón	91.594	Leather	Cuero	72.754
Garlic	Ajo	91.304	Pear	Pera	72.174
Banana	Plátano	89.855	Carrot	Zanahoria	67.826
Bread	Pan	89.275	Mustard	Mostaza	66.667
Cigarette	Tabaco	87.536	Chamomile	Manzanilla	66.377
Peppermint	Menta	86.667	Walnut	Nuez	62.029
Cinnamon	Canela	86.667	Cherry	Cereza	61.739
Strawberry	Fresa	85.507	Plum	Ciruela	60.870
Pineapple	Piña	85.507	Fir	Abeto	58.551
Ham	Jamón	85.507	Blackberry	Mora	58.261
Smoke	Humo	85.217	Raspberry	Frambuesa	54.493
Anise	Anís	84.638	Rum	Ron	54.203
Rose	Rosa	84.058	Clove	Clavo	52.464
Apple	Manzana	83.768	Grapefruit	Pomelo	51.304
Melon	Melón	83.768	Chive	Cebollino	51.014
Grass	Césped	82.899	Sauerkraut	Coles de Bruselas	50.725
Spearmint	Hierbabuena	82.029	Rubber	Caucho	49.275
Glue	Pegamento	81.159	Chamomile	Camomila	46.667
Peach	Melocotón	81.159	Trementine	Trementina	42.609
Wine	Vino	80.870	Sauerkraut	Chucrut	37.971
Solvent	Disolvente	79.125			

**Table 2:** Normative values per age group of OT, OD, OI, and TDI.

		OT	OD	OI	TDI
**Overall sample**					
N		209	209	209	209
Mean		6.37	10.57	12.82	29.76
SD		2.66	2.48	2.18	4.8
Mean CI 95%		(6.01, 6.73)	(10.23, 10.9)	(12.52, 13.11)	(29.11, 30.4)
Min		0	0	5	12.5
Max		14.5	16	16	41
Percentiles	5	1.5	6	8	20.75
	10	3	7	10	22.5
	25	4.5	9	12	27.25
	50	6.25	11	13	30.5
	75	8.25	12	14	33.12
	90	9.75	13	15	35
	95	11.12	14	16	36.62
**Age group (20–30)**					
N		33	33	33	33
Mean		8.17	11.15	13.00	32.35
SD		2.28	2.37	2.02	3.90
Mean CI 95%		(7.39, 8.94)	(10.34, 11.96)	(12.3, 3.69)	(31.01, 33.68)
Min		3.25	6	8	22.5
Max		14.5	16	13	41
Percentiles	5	4.8	8	10	26.5
	10	5.65	8	11	27.8
	25	6.5	10	12	29.5
	50	8.5	11	13	32.5
	75	9.5	13	15	34.5
	90	10.6	13.8	16	37.45
	95	11.8	14.8	16	38.25
**Age group (30–40)**					
N		25	25	25	25
Mean		7.47	12.04	13.76	33.23
SD		2.47	1.86	1.50	2.46
Mean CI 95%		(6.5, 8.44)	(11.31, 12.77)	(13.17, 14.35)	(32.27, 34.19)
Min		2.5	9	11	27.5
Max		12.5	15	16	39
Percentiles	5	3.35	9	11.2	29.6
	10	5.05	9	12	30.2
	25	6.25	11	13	31.5
	50	7.5	12	14	33.5
	75	8.5	13	15	34.5
	90	11	14	15.6	35.3
	95	11.4	14.8	16	36.5
**Age group (40–50)**					
N		29	29	29	29
Mean		6.61	11.20	13.48	31.30
SD		2.53	2.02	1.57	3.54
Mean CI 95%		(5.69, 7.53)	(10.47, 11.94)	(12.91, 14.05)	(30.01, 32.58)
Min		2.5	6	10	23.5
Max		11.5	14	16	38.5
Percentiles	5	3.5	8	11	25.9
	10	4.1	8.8	11.8	27.3
	25	4.5	10	12	28.5
	50	6	12	14	31.25
	75	8.5	13	14	33.5
	90	10.5	13	15.2	35.5
	95	10.65	13.6	16	37.3
**Age group (50–60)**					
N		71	71	71	71
Mean		6.01	10.27	13.11	29.38
SD		2.58	2.38	1.84	4.10
Mean CI 95%		(5.41, 6.61)	(9.71, 10.82)	(12.68, 13.54)	(28.43, 30.33)
Min		1.5	5	6	17.5
Max		13.25	16	16	38.5
Percentiles	5	1.5	6.5	11	22.5
	10	3.5	7	11	24.5
	25	4.5	8.5	12	26.5
	50	5.5	10	13	29.5
	75	7.5	12	14.5	32.5
	90	9.25	13	15	33.5
	95	10.5	14.5	16	36
**Age group (>60)**					
N		51	51	51	51
Mean		5.01	9.53	11.45	26.03
SD		2.35	2.69	2.93	5.13
Mean CI 95%		(4.37, 5.65)	(8.79, 10.27)	(10.64, 12.26)	(24.62, 27.44)
Min		0	0	4	12.5
Max		11.25	14	15	30.25
Percentiles	5	1.5	6	5	17.375
	10	1.5	6	7	20.5
	25	3.5	7.5	10.5	22.5
	50	5.5	10	12	27.25
	75	6.375	11	13.5	30
	90	7.5	13	15	31.5
	95	8.875	13	15	33

SD = standard deviation, CI = confidence interval, Min = minimum, Max = maximum.

**Table 3:** Internal consistency and test−retest reliability.

	OT	OD	OI	TDI
**Test-retest reliability (*n* = 22)**				
Mean concordance correlation ^a^	0.45	0.44	0.66	0.56
Intraclass correlation	0.49 *	0.48 *	0.69 **	0.6 **
Pearson’s correlation	0.51 *	0.48 *	0.69 **	0.6 **
**Internal consistency (*n* = 209)**				
Cronbach’s alpha	0.95	0.46	0.53	0.4
Spearman−Brown coefficient	0.95	0.43	0.46	0.4

* *p* < 0.05, ** *p* < 0.01. ^a^ No inferential test is performed. CI = Confidence interval.

## Supplementary Materials

The following are available online at https://www.mdpi.com/2076-3425/10/12/943/s1, Table S1: Descriptive statistics of OT, OD, OI and TDI scores per Sex and Age.
