# Supplementary material for: Spanish Validation for Olfactory Function Testing Using the Sniffin’ Sticks Olfactory Test: Threshold, Discrimination, and Identification
Source: Brain Sci. 2020 Dec 7;10(12):943. doi: 10.3390/brainsci10120943 (PMC7762307; doi:10.3390/brainsci10120943)
Supplement: Supplementary file 1 [file brainsci-10-00943-s001.pdf]

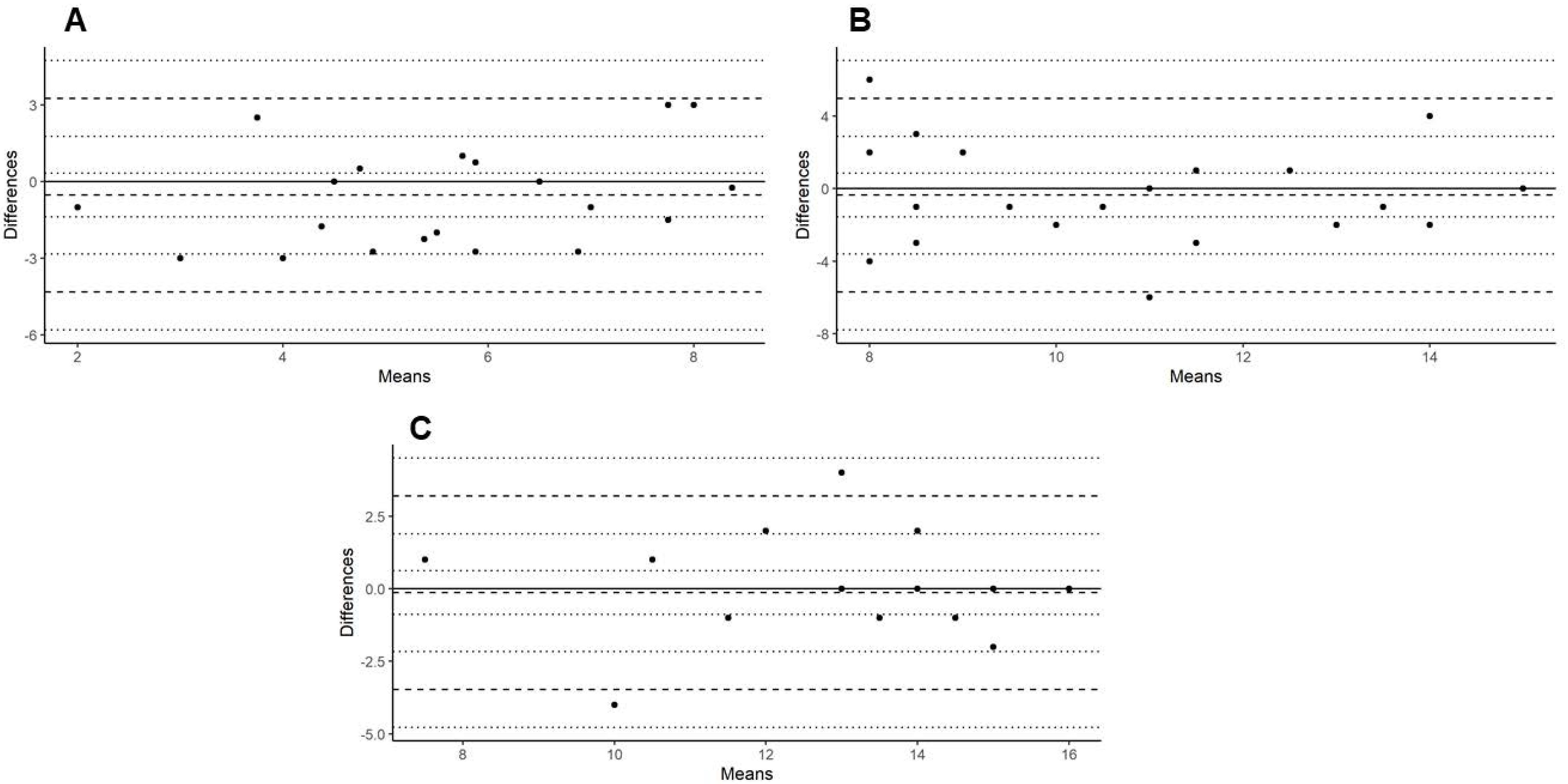

Supplementary Figure S1. Bland-Altman plots for olfactory subtests. A) Olfactory Threshold (OT). B) Olfactory Discrimination (OD). C) Olfactory Identification (OI).
